# Creative dance associated with traditional Portuguese singing as a strategy for active aging: a comparative cross-sectional study

**DOI:** 10.1186/s12889-022-12978-4

**Published:** 2022-03-29

**Authors:** Paulo Coelho, José Marmeleira, Ana Cruz-Ferreira, Luís Laranjo, Catarina Pereira, Jorge Bravo

**Affiliations:** 1 Departamento de Desporto E Saúde, Escola de Saúde E Desenvolvimento Humano, Universidade de Évora, Évora, Portugal; 2 Comprehensive Health Research Center (CHRC), Universidade de Évora, Évora, Portugal

**Keywords:** Expressive movement, Singing, “Cante Alentejano”, Physical fitness, Older adults

## Abstract

**Background:**

Multimodal forms of exercise can influence several physical and mental factors important for successful aging. In the present study, we introduce a new type of multimodal intervention, combining movement (creative dance) with traditional singing. This study aims to compare physical fitness, functional physical independence, depressive symptoms, general cognitive status, and daytime sleepiness among older adults participating in multimodal exercise, those participating in traditional physical exercise, and those not actively engaged in physical exercise.

**Methods:**

This cross-sectional study included 112 people aged ≥ 65 years (75.3 ± 0.7 years) living independently in the community, divided into 3 groups: multimodal exercise (*n* = 34), traditional exercise (*n* = 41), and no physical exercise (*n* = 37).

**Results:**

The multimodal exercise group showed greater flexibility of the lower limbs and upper limbs/shoulders and better general cognitive status than the traditional exercise group (*p* < 0.05). The traditional exercise group had better agility and dynamic balance, aerobic endurance, and strength of the lower and upper limbs than the no-exercise group (*p* < 0.05).

**Conclusions:**

The results suggest that the two types of programs studied may have different impacts on some of the variables investigated and support the design of future experimental studies that include interventions based on the combination of creative dance and traditional Portuguese singing.

## Background

Aging is a complex and multifactorial process that involves several biological, physiological, lifestyle, and environmental factors [1]. The aging process is different between people and varies based on genetics, lifestyle, and the accumulation of different life experiences [2]. Physical exercise is one of the most commonly prescribed interventions to minimize the detrimental effects of aging and maximize the health, quality of life, and autonomy of older people [3, 4].

Physical exercise promotes functional fitness, including muscle strength, flexibility, cardiorespiratory capacity, and balance [5]. In addition, it improves the quality of gait and reduces the risk of falls, resulting in greater efficiency in the performance of daily activities and positive changes in the quality of life of older people [6, 7]. Physical exercise is also associated with improvements in the cognitive, social, and emotional domains [8, 9]. Several psychological aspects benefit from exercise participation, including mood states, depression, anger, confusion, vitality, stress, self-esteem, self-concept, depression, and anxiety [10]. Furthermore, exercise can also facilitate social interactions and prevent isolation among older people [11].

Dance is a specific form of exercise that occurs in pleasant and energetic environments [12, 13] and contributes to successful aging [12, 14]. Dance can improve motor skills, balance, coordination, aerobic endurance, social relationships, and positive emotions [12, 14–17]. In particular, creative dance has been shown to improve proprioception [18], strength, aerobic endurance, flexibility, agility and dynamic balance, body composition, and life satisfaction [19]. Recently, a creative dance program was effective in improving the strength, flexibility, functional balance, and mobility of older adults [12].

Participation in artistic and cultural activities has also been used to promote the well-being, quality of life, and health of older people [20, 21]. Among these activities, singing has attracted considerable interest in the scientific community. There is evidence of its positive effects on personal and social well-being, cognitive functioning, and physiological health [20, 22]. A study with older people belonging to a choral singing group showed benefits of singing in three quality of life domains (psychological, social relationships, and environment), regardless of age and the presence of depressive symptoms [23]. A qualitative study also reported that singing activities could have a positive influence on social, emotional, physical, and cognitive domains irrespective of age, gender, nationality, or well-being status [24].

The present article introduces a novel type of multimodal exercise, combining movement (creative dance) with traditional singing. The singing component of the intervention is based on “Cante Alentejano”, a traditional polyphonic singing endogenous to the Alentejo region (Portuguese region where the current study was developed), in which the lyrics explore rural life themes [25]. "Cante Alentejano" is part of the Intangible Cultural Heritage of Humanity of United Nations Educational, Scientific and Cultural Organization (UNESCO). For comparion, we include another type of exercise in the present study, which we called "traditional exercise" as it includes common features of exercise for older adults, namely, aerobic, strength, balance, agility, and range-of-motion exercises.

Furthermore, in the present study, we consider several health and functional indicators that influence older adults' quality of life, namely, functional physical fitness, functional physical independence, depressive symptoms, general cognitive status, and daytime sleepiness. This study aims to compare these health indicators among older adults participating in multimodal exercise (creative dance combined with traditional Portuguese singing), those participating in traditional physical exercise, and those not actively engaged in physical exercise.

## Methods

### Study design and participants

This comparative cross-sectional analytical study included older adults ≥ 65 years of age (75.3 ± 0.7 years) living independently in the community. Participants were recruited in Évora and Beja districts (Portugal) through a direct invitation in social centers, pensioners' associations, senior universities, and parish centers.

**Fig. 1:**
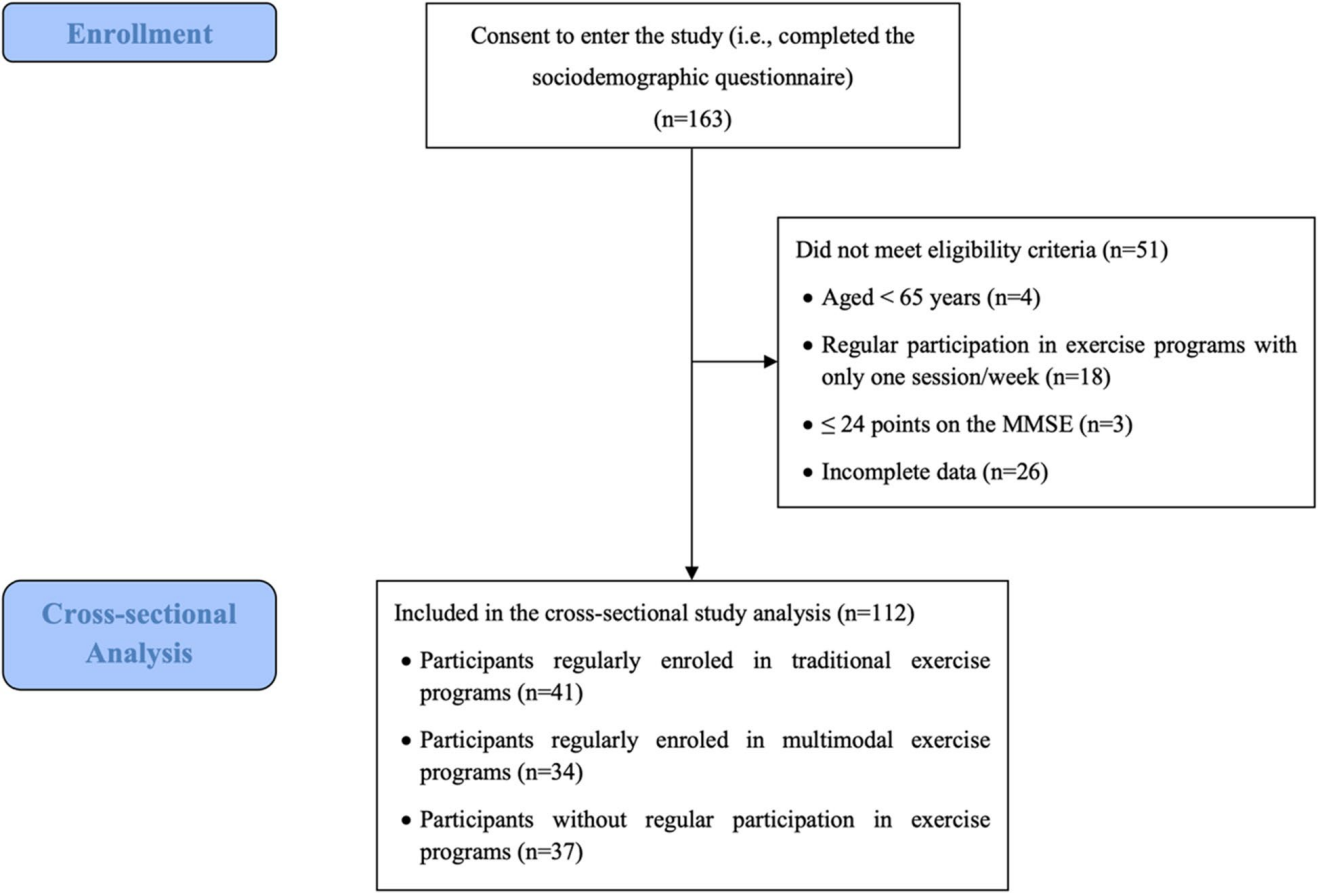
Flow Diagram

The inclusion criteria were as follows: ≥ 65 years of age, absence of cognitive impairment [> 24 points on the Folstein Mini-Mental State Examination (MMSE)] [26] and the ability to autonomously carry out the exercise sessions and physical evaluations. For the participants involved in physical exercise programs, only those who performed traditional exercise or multimodal exercise (creative dance combined with traditional Portuguese singing) twice a week for at least six months were considered for the present study. The participants included in the no-exercise group could not have been engaged in any physical exercise program in the last six months before data collection. In total, a sample of 112 older people was assembled and distributed as follows (Fig. 1): traditional exercise group (TEG) with 41 participants (77.8 ± 1.2 years old), multimodal exercise group (MEG) with 34 participants (74.1 ± 1.1 years of age), and no-exercise group (NEG) with 37 participants (73.9 ± 1.1 years of age). This study was approved by the University of Évora Ethics Committee for research in human health and well-being (reference number 16–012), having followed the Declaration of Helsinki. All participants gave their written consent to participate in this study.

### Procedures

Between April 2017 and January 2018, the evaluations took place in appropriate rooms for physical exercise practice (Beja and Évora). The tests were applied under the same conditions. Two raters were trained to apply the tests, which revealed interrater reliability between 0.722 and 0.999 as assessed through Pearson and Spearman's bivariate correlation coefficients.

The participants were given instructions to perform the tests, reinforced with demonstrations, and asked to do their best in each test. Additionally, individual questionnaires were administered to all participants in a calm and safe environment in the form of an interview. This questionnaire was used to collect sociodemographic information.

### Instruments

#### Anthropometry and physical activity levels

The participants were weighed on an appropriate scale (Seca, Hamburg, Germany) to the nearest 0.1 kg, wearing minimal clothing and barefooted. Height was measured to the nearest 0.1 cm, according to standardized procedures [27] with a scale attached to a stadiometer (Seca, Hamburgo, Alemanha). The body mass index (kg/m^2^) was calculated from the weight (kg) divided by the height (m) squared.

Waist circumference was measured according to the World Health Organization protocol [28] with anthropometric tape (Seca, Hamburg, Germany). The average of two measurements was calculated, and if they differed by more than 1.0 cm, a third measurement was made, averaging the two closest measurements.

Habitual physical activity levels and sedentary behavior were evaluated to quantify a possible interference of physical activity in the study's dependent variables and measured with the International Physical Activity Questionnaire [29]. The total metabolic expenditure (MET-min/week) was calculated by determining the time (min/day) and frequency (days/week) spent in each of these activities.

#### Functional physical fitness and balance

Functional fitness included the assessment of agility and dynamic balance (time in seconds in the 8-foot up-and-go test), lower limb flexibility (distance in centimeters in the chair sit-and-reach test), upper limb/shoulder flexibility (distance in centimeters in the back scratch test), lower body strength (number of repetitions in the chair stand test), upper limb strength (number of repetitions in the arm curl test) and aerobic endurance (distance covered in meters in the 6-min walk test) of the Senior Fitness Test battery [30]. Dynamic and static balance was assessed using the Fullerton Advanced Balance scale [31], composed of ten tasks rated on an ordinal scale between 0 (worst) and 4 points (best). The sum of the points obtained in each of the ten tasks corresponded to the final grade in the multidimensional balance measure (0–40 points).

#### Physical and functional independence, depressive symptoms, general cognitive status and daytime sleepiness

Physical and functional independence was measured using the Composite Physical Function scale [32], which includes 12 daily activities divided into ten instrumental activities (related to community tasks, such as housework or commuting) and two basic activities (related to personal functioning, such as hygiene and nutrition). The evaluation of depressive symptoms was carried out using the Portuguese version of the Geriatric Depression Scale [33, 34]. General cognitive status was assessed using the MMSE [26, 35], with an internal structure of 20 individual tests assessing the nomination, repetition, three-step command, reading, writing a sentence, copying intersecting pentagons, orientation, attention/concentration/calculation, memory and recall domains. The Epworth Sleepiness Scale (ESS) [36] was used to assess daytime sleepiness, a scale consisting of eight items, where participants were asked to rate the probability of falling asleep in eight different daily life situations on an increasing scale (0–3 points).

#### Exercise programs

Both exercise programs (MEG and TEG) were carried out by specialized and accredited instructors (graduated in physical education or sports sciences). The physical exercise session instructors were blind to the study objectives and had not participated in the data collection or analysis. Exercise program uniformity was guaranteed for each group through the joint planning of the exercise sessions. The participants included in the TEG and MEG were involved in the respective exercise program for more than six months, with two weekly sessions lasting 50 min each.

The exercise program for the TEG focused on improving motor and coordination skills to enhance functional performance. Exercise sessions started with a warm-up period (approximately 10 min) that included light mobility exercises for cardiorespiratory and neuromuscular activation. The exercises consisted of walking in different directions with changes in the speed of movement and range of stride length and simple games of movement that involved touching, catching, throwing, or swinging. Dynamic muscle stretching and joint mobilization exercises were also performed. With a duration of 35 min, the main part of the session involved the stimulation of various physical capacities (flexibility, agility, dynamic balance, strength, and aerobic endurance) that alternated across in six mandatory stations. At each station (approximately 5 participants per station), the exercises were aimed to be adapted and individualized at an intensity based on the participant’s characteristics for 3 min, with 2 min of active recovery between rotations. In the last 5 min, a return to rest was accomplished by breathing exercises and stretching the main muscle groups with the aim to reduce muscle and joint tension. The intensity of the tasks was gradually increased throughout the exercise program, while considering each participant’s individuality and specificity.

The MEG program sessions started with a warm-up period (10 min) involving a slight, low-pitched vocal warm-up (inhalation, exhalation, yawning movement, lip, and tongue vibration). Simultaneously, the participants performed simple movements with different degrees of balance, coordination, and flexibility, while exploring the free space, following the instructors' indications (e.g., static stretching movements, creative dance games, walking forward, backward, to the right or left, slow/fast gait, alternating steps with hands-on-hips, alternating steps and leg flexion–extension with hands-on-hips, lifting the knees, and arms extension-flexion), and synchronizing the expressive movements of creative dance with traditional Portuguese singing. In the main part of the session, with a duration of 35 min, the participants worked in groups (organized in different spatial formations such as circle, quadrille, and columns). In this phase of the session, the participants simultaneously sang traditional Portuguese music and explored the expressive dance movements, while following the instructor’s visual demonstration. Although there was imitation work, the participants were encouraged to explore their movement from the basic elements of movement: body (body parts), space (formations, directions, levels, trajectories), time (slow/fast/pause), dynamics (strong/weak/straight/wavy) and interrelationships (pairs and in groups). Songs such as “ceifeira”, “monda”, “ciranda”, “pastor”, “ó rama da oliveira”, “parreira”, “romã”, “ribeira vai cheia”, and “gotinha de água”, among others, were examples of the type of singing that was used, and the lyrics of the songs were used as a stimulus for creating movement. The activities carried out in the sessions evolved from simple and light tasks to more complex and moderate tasks. At the end of the session (5 min), some muscle stretching exercises were performed, working on relaxation, especially breathing exercises.

#### Data analysis

Since the participants' demographic and anthropometric variables did not follow a normal distribution, differences between groups were analyzed using Mann–Whitney *U* tests. The comparisons among the three groups were made through successive covariance analyses (ANCOVA) with adjustments for each group’s average age and sex proportions.

Additionally, Cohen's d was calculated for the variables under study, with 90% confidence intervals to measure the magnitude of the differences between the groups. The effect size limits were considered as follows: no effect (d < 0.2), small effect (0.2 ≤ d < 5), medium effect (≤ 0.5 d < 0.8) or large effect (d ≥ 0.8) [37]. The significance level of *p* < 0.05 was used for all statistical tests performed. Data analysis was performed using SPSS Statistics software (version 22.0 for Windows; IBM), and the effect sizes were calculated online (http://www.socscistatistics.com/effectsize/Default3.aspx).

## Results

The participant sociodemographic profile is presented in Table 1. There were no significant differences in weight, body mass index, or abdominal circumference among the participants in the three groups. Regarding participant height, the results revealed significant differences between the NEG and MEG (*p* = 0.013) and between the NEG and TEG (*p* = 0.031). Physical activity levels did not significantly differ among the groups.

**Table 1:** Sociodemographic characteristics of the participant

Variables	NEG (*n* = 41)	TEG (*n* = 42)	MEG (*n* = 36)
Age (years)	77.8 ± 1.2	73.9 ± 1.1 ^a^	74.1 ± 1.1 ^b^
Sex (women/men)	29/12	38/4	33/3
Height (cm)	156.5 ± 7.1	152.8 ± 8.7 ^a^	153.1 ± 5.3
Weight (kg)	67.9 ± 11.7	67.5 ± 12.8	70.2 ± 12.1
BMI (kg/m^2^)	27.9 ± 4.3	28.7 ± 4.0	29.71 ± 4.5
Waist circumference (cm)	97.7 ± 11.7	94.9 ± 10.9	97.9 ± 10.6
Physical activity (MET/min/week)	1061.1 ± 1468.7	1407.2 ± 1718.3	2323.2 ± 2643.6

Values represent mean ± standard deviation. *NEG* no-exercise group, *TEG* traditional exercise group, *MEG* multimodal exercise group, *BMI* body mass index, *cm* centimeters; *kg* kilograms; *kg/m*^*2*^ kilograms per square meter, *MET/min/week* MET per minute per week. ^a^ Significant difference between NEG and TEG (*p* < 0.05). ^b^ Significant difference between NEG and MEG (*p* < 0.05)

**Fig. 2:**
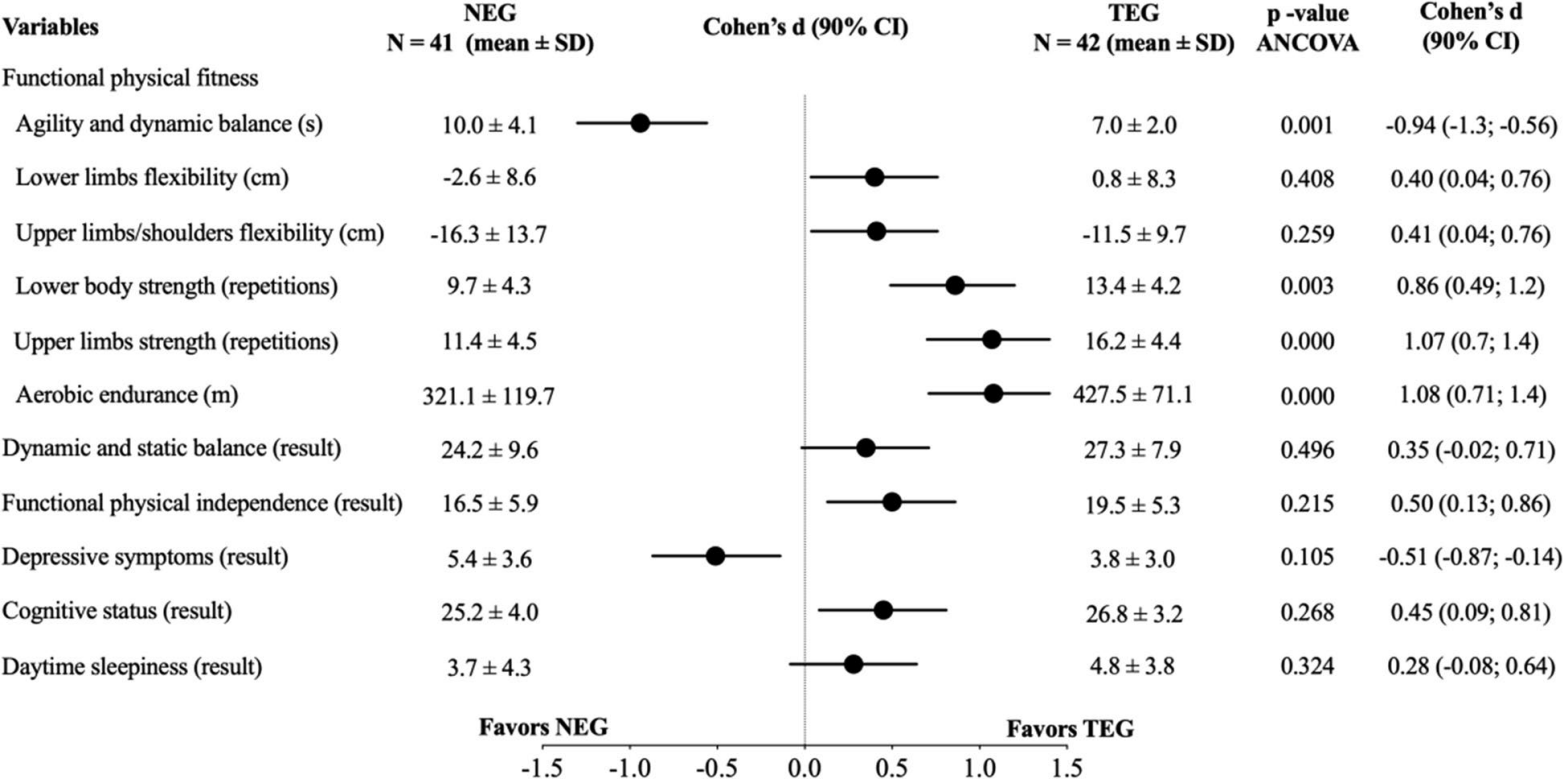
The comparison between the NEG and the TEG, with adjustment to each group average age and sex proportions

Figure 2 shows the comparative results between the NEG and TEG, with adjustments for the average age of each group. The NEG participants showed less agility and dynamic balance than the TEG participants (*p* = 0.001; large effect: d = -0.94). The TEG participants also had higher strength levels in the lower body and upper limbs (*p* = 0.003, large effect: d = 0.86; *p* = 0.000, large effect: d = 1.07, respectively) and greater aerobic endurance (*p* = 0.000; large effect: d = 1.08).

**Fig. 3:**
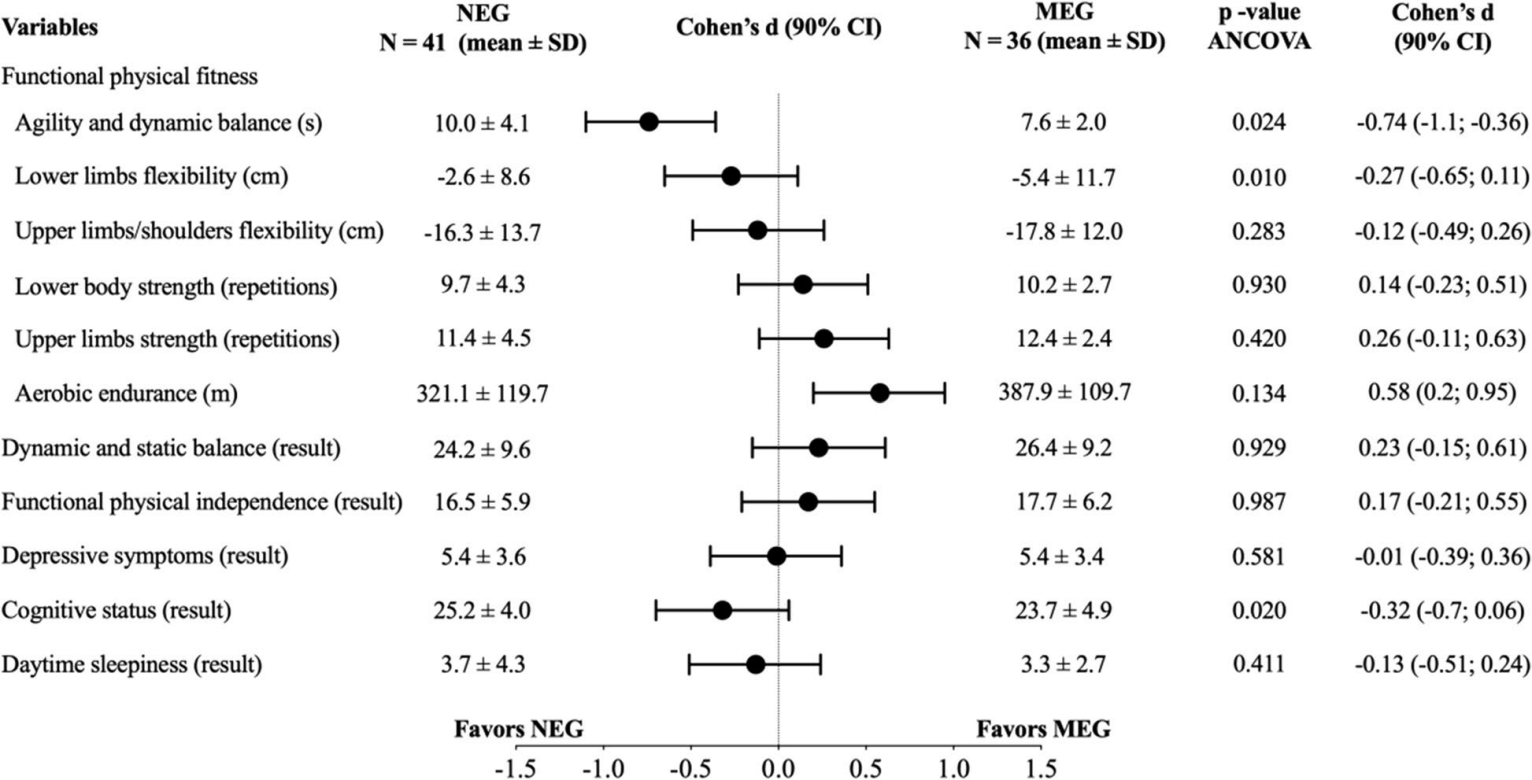
The comparison between the NEG and the MEG, with adjustment to each group average age and sex proportions

Comparing the NEG with the MEG (Fig. 3), maintaining the adjustment for the average age of each group, the MEG participants showed greater agility and dynamic balance than the NEG participants (*p* = 0.024; average effect: d = -0.74), greater flexibility of the lower limbs (*p* = 0.010) and a more favorable general cognitive status (*p* = 0.020), despite a small effect for the latter two (d = -0.27; d = -0.32, respectively).

**Fig. 4:**
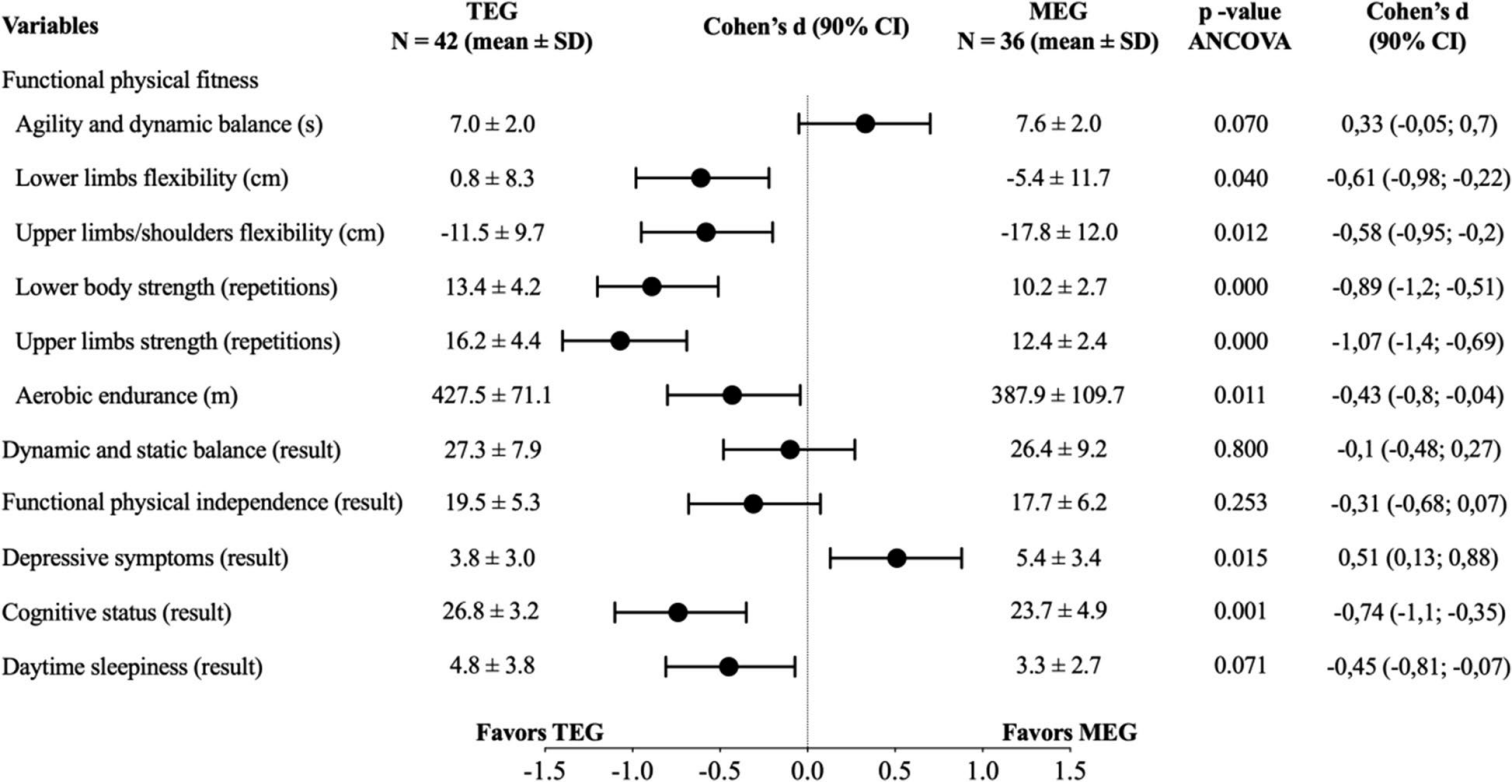
The comparison between the TEG and the MEG, with adjustment to each group average age and sex proportions

Figure 4 shows the comparative results between the two groups involving physical exercise (TEG and MEG) with adjustments for the average age of each group. The MEG participants showed greater flexibility of the lower limbs and upper limbs/shoulders than TEG participants (*p* = 0.040, average effect: d = -0.61; *p* = 0.012, average effect: d = -0.58, respectively). On the other hand, for both lower limb (*p* = 0.000; large effect: d = -0.89) and upper limb strength (*p* = 0.000; large effect: d = -1.07) and aerobic endurance (*p* = 0.011), the MEG participants had worse results than the TEG participants, although with a small effect (d = -0.43) for aerobic endurance values. The MEG participants also had a more favorable general cognitive status (*p* = 0.001; mean effect: d = -0.74) than TEG participants, although they had more depressive symptoms (*p* = 0.015; mean effect: d = 0.51).

## Discussion

The present study aimed to compare health indicators between older adults participating in multimodal exercise (creative dance combined with traditional Portuguese singing), those participating in traditional physical exercise, and those not actively engaged in physical exercise. To our knowledge, this is the first study showing the potential benefits of multimodal exercise based on creative dance practice in association with the interpretation of traditional Portuguese songs, especially when comparing the results of regular practitioners of this type of exercise with individuals not engaging in physical exercise. Regular practice of multimodal exercise, which combined creative dance with traditional Portuguese singing, seemed to result in better agility and balance, flexibility, and general cognitive status. The practice of traditional physical exercise has also been shown to be beneficial compared to the absence of regular physical exercise, revealing that participants in these types of programs have greater agility and balance, higher rates of strength and aerobic endurance. In the comparison of this multimodal exercise with traditional exercise, both showed benefits in different conditions, with multimodal exercise practitioners showing higher benefits in flexibility and general cognitive status. In contrast, traditional exercise practitioners showed greater benefits in strength, aerobic endurance, and depressive symptoms. However, both groups were below the diagnostic values of depression [33].

Traditional physical exercise programs, mostly multidimensional by design, aim to develop strength, balance, aerobic endurance, and flexibility [38]. This type of intervention has proven to be effective in promoting healthy aging, mainly due to the beneficial effects that it produces on balance, strength, and aerobic endurance. These improvements have direct consequences in carrying out daily tasks and reducing the risk of falls in older people [6, 7, 39, 40]. In the present study, the participants in traditional physical exercise programs showed results consistent with previous interventions [6, 7, 39, 40], although it was a cross-sectional study.

In older people, when intervention groups involving physical exercise are compared to control groups, even in randomized controlled studies, it is common to register significant differences between exercise groups and control groups, regardless of the type of exercise performed [6]. In addition to the benefits registered in the present study’s TEG participants, the MEG participants, where creative dance was combined with traditional Portuguese singing, showed better rates of agility and balance, flexibility, and a more favorable general cognitive status compared to the NEG. Some interventions that include physical exercise practice based on dance were shown to be effective in improving balance and gait and were considered by the participants as a pleasant form of exercise [41]. However, Cruz-Ferreira et al. [19] found that physical exercise specifically focused on creative dance revealed beneficial effects of this practice on lower limb strength in 21% of practitioners, lower limb flexibility, agility, and dynamic balance in 13%, aerobic endurance in 10%, waist circumference in 8%, body mass index in 5% and satisfaction with life in 34%. In the same manner, other scientific research reported beneficial effects of dance practice not only on promoting balance, proprioception, strength, flexibility, posture, and aerobic endurance but also on improving social relationships and promoting positive emotions, thereby contributing to healthy aging [14–18]. Benefits from dance practice seem to come from the stimulation of brain functions related to memory, cardiovascular stimulation, cognitive stimulation, and the social and emotional interactions promoted in this type of activity [14].

Comparing usual practitioners of traditional exercise and practitioners of multimodal exercise in the present study revealed higher levels of flexibility and a better general cognitive status for the multimodal exercise practitioners, who engaged in creative dance with traditional Portuguese singing. In the meantime, the practitioners of traditional physical exercise revealed higher strength and aerobic endurance levels and fewer depressive symptoms. A relatively recent study with 30 sedentary women between the ages of 60 and 75 living in the community compared regular dance practitioners (3x/week, 60-min sessions) with walkers and stretching practitioners in an intervention that lasted eight weeks [42]. Dancing induced similar improvements to walking in cardiorespiratory fitness, lower limb muscle strength, and static balance. In addition, the practice of dance associated with traditional Portuguese singing seemed to induce brain function stimulation related to memory and influenced practitioners’ general cognitive status, as suggested by other authors [14].

This is the first study to analyze a group of subjects in a multimodal exercise program that combines creative dance and traditional Portuguese singing to refer the practitioner to their previous experiences and cultural heritage while making body expression movements (adjusted and structured spontaneous exercises) compared to a group of people engaged in a traditional exercise program. The results of the present study support using this form of physical exercise as a strategy to promote healthy aging. Thus, it creates the foundation for performing random controlled interventions to prove the effects of multimodal exercise combining expressive dance and traditional Portuguese singing.

Findings from our study should be interpreted with caution due to its cross-sectional design. We consider this to be one of its main limitations since it does not establish cause-effect relationships. Even so, we believe that our findings can guide future randomized controlled trials and providing indications for the potential of multimodal exercise that combines expressive dance and traditional Portuguese singing. Therefore, longitudinal studies are needed to confirm the beneficial effects of our multimodal exercise approach. Another limitation is related to the fact that the intensity of the physical exercise sessions was not monitored and should be considered an essential requirement in future research.

## Conclusion

This study found that the regular practice of creative dance, combined with traditional Portuguese singing, is associated with greater agility and balance, flexibility, and general cognitive status in older adults. The present study results suggest that this form of physical exercise seems to induce different and complementary benefits to the practice of traditional physical exercise for this population. These results provide evidence for creating physical exercise programs based on the association between creative dance and traditional Portuguese singing to promote healthy aging.

## Data Availability

The datasets used and/or analyzed during the current study are available from the corresponding author upon reasonable request.

## Abbreviations

MMSE: Mini-Mental State Examination
TEG: Traditional Exercise Group
MEG: Multimodal Exercise Group
NEG: No-Exercise Group
MET: Metabolic Equivalent of Task
ESS: Epworth Sleepiness Scale
ANCOVA: Covariance Analyzes
SPSS: Statistical Package for the Social Sciences
SD: Standard Deviation
CI: Confidence Intervals
s: Seconds
cm: Centimeters
m: Meters
